# A Statistical Similarity/Dissimilarity Analysis of Protein Sequences Based on a Novel Group Representative Vector

**DOI:** 10.1155/2019/8702968

**Published:** 2019-05-08

**Authors:** Marwa A. Abd Elwahaab, Mervat M. Abo-Elkhier, Moheb I. Abo el Maaty

**Affiliations:** Department of Engineering Mathematics and Physics, Faculty of Engineering, Mansoura University, Mansoura 35516, Egypt

## Abstract

Similarity/dissimilarity analysis is a key way of understanding the biology of an organism by knowing the origin of the new genes/sequences. Sequence data are grouped in terms of biological relationships. The number of sequences related to any group is susceptible to be increased every day. All the present alignment-free methods approve the utility of their approaches by producing a similarity/dissimilarity matrix. Although this matrix is clear, it measures the degree of similarity among sequences individually. In our work, a representative of each of three groups of protein sequences is introduced. A similarity/dissimilarity vector is evaluated instead of the ordinary similarity/dissimilarity matrix based on the group representative. The approach is applied on three selected groups of protein sequences: beta globin, NADH dehydrogenase subunit 5 (ND5), and spike protein sequences. A cross-grouping comparison is produced to ensure the singularity of each group. A qualitative comparison between our approach, previous articles, and the phylogenetic tree of these protein sequences proved the utility of our approach.

## 1. Introduction

Sequence comparison is used to study structural and functional conservation and evolutionary relations among the sequences. The importance of similarity/dissimilarity of biological sequences returns to its relationship with the structures and functions. Proteins with similar sequences usually have similar structures. The rate of addition of new sequences to the databases is increasing exponentially [1]. Comparing these new sequences to those with known functions is a key way of understanding the biology of an organism. Thus, sequence analysis can be used to assign function to genes and proteins by the study of the similarities between the compared sequences. There are many tools and techniques that provide the sequence comparisons.

Sequence comparison can be classified into alignment-based methods and alignment-free methods [2, 3]. Alignment-based methods assign scores to different possible alignments, picking the alignment with the highest score. Some algorithms do global alignment or local alignment [4–6]. BLAST [7] and FASTA [8] are the most widely used applications. Alignment-based methods are computationally difficult with multiple sequence alignments at the same time. A wide range of scoring systems has been proposed such as amino acid substitution scoring matrices PAM and BLOSUM for protein alignment [9].

Alignment-free approaches overcome the limitations of alignment-based methods. Graphical representation approaches are one of them. Graphical representations are usually accompanied by numerical characterization and then a descriptor to describe each protein sequence. A similarity/dissimilarity analysis is then done using these descriptors by evaluating Euclidean distance or correlation angle among them. The smallest Euclidean distance or correlation angle is the more similar. Many graphical representations of DNA and protein primary sequences have been proposed. Some other approaches characterize numerically protein sequences without previous graphical representation and nongraphical representation methods [10, 11].

In this article, an alignment-free method is introduced. It is considered a nongraphical representation method. Three groups of protein sequences are selected to illustrate our approach. They are beta globin, NADH dehydrogenase subunit 5 (ND5), and spike protein sequences. They are selected as each group has sequences of similar range of lengths. The most common sequences of each group are selected. The selected sample is assumed to be unbiased and the population distribution of each group is normal. Therefore, the selected sample represents the group. Statistics can be used to estimate the population's parameters. The adjacency vector is introduced as a novel descriptor for protein sequences. It is computed for each sequence in the selected sample of three groups. A reference vector is then computed for each group. This vector acts as a representative of the group. Each sequence's degree of similarity in each group is measured according to its group's representative vector. So, a similarity/dissimilarity vector is constructed instead of ordinary similarity/dissimilarity matrix. Our approach is independent of the protein sequence length. It does not require any previous graphical representation. It is a mathematically simple approach.

## 2. Dataset, Technology, and Tools

The protein sequences used in this article are listed in Tables 1, 2, and 3. The sequences are downloaded from the National Center for Biotechnology Information (NCBI) “https://www.ncbi.nlm.nih.gov/” as FASTA files. These FASTA files are imported into Wolfram Mathematica 8 where all the results and figures are produced. The phylogenetic tree of these protein sequences is also created by the Basic Local Alignment Search Tool (BLAST) “https://blast.ncbi.nlm.nih.gov/Blast.cgi”.

Table 1 shows the 1^st^ sample set that consists of seven species of beta globin protein sequences. Their range of lengths is from 121 to 147. This sample set is applied before in [12].  Table 2 shows the 2^nd^ sample set which consists of nine ND5 protein sequences. Their range of lengths is from 602 to 610. This sample set is applied before in [12–25].  Table 3 shows the 3^rd^ sample set which consists of 29 spike protein sequences. Their range of lengths is from 1162 to 1447. These viruses are coronavirus. They are classified into four classes: Class I that includes the porcine epidemic diarrhea virus (PEDV) and the transmissible gastroenteritis virus (TGEV). Class II includes the bovine coronavirus (BCoV), human coronavirus OC43 (HCoV-OC43), and the murine hepatitis virus (MHV). Class III contains the infectious bronchitis virus (IBV). The others are severe acute respiratory syndrome coronaviruses (SARS-CoV). This sample set is applied before in [26].

## 3. The Adjacency Vector

In this approach, a new vector is suggested to be a descriptor of a protein sequence. This vector is called the adjacency vector (*A*_*xy*_);* x* refers to the species' protein sequence and* y* refers to its related group. It counts the occurrence of all possible pairwise adjacencies obtained by reading the protein primary sequence from left to right. The protein sequence is composed of 20 common different amino acids which are “A,” “R,” “N,” “D,” “C,” “Q,” “E,” “G,” “H,” “I,” “L,” “K,” “M,” “F,” “P,” “S,” “T,” “W,” “Y,” and “V” as ordered alphabetically according to 1^st^ letter code. Therefore, the adjacency vector (*A*_*xy*_) consists of 400 elements. Every 20 elements are related to each amino acid. The first 20 elements are related to “A” amino acid. The second 20 elements are related to “R” amino acid. The third 20 elements are related to “N” amino acid and so on by the same order which is illustrated previously according to 1^st^ letter code. We borrow our idea from the 20 ×20 adjacency matrix [27].

The adjacency vector counts the possibilities of each pair. In other words, it counts the number of times that each pair is repeated along the sequence length. If the pair does not exist, its value in the adjacency vector is zero. For example, to evaluate the adjacency vector of the two short segments of “yeast Saccharomyces cerevisiae” protein [16, 19, 22–24, 28]

Protein I: “WTFESRNDPAKDPVILWLNGGPGCSSLTGL”

Protein II: “WFFESRNDPANDPIILWLNGGPGCSSFTGL”

The two protein sequences are composed of 30 amino acids. Protein I is converted to 29 adjacent pairs that are WT, TF, FE, ES, SR, RN, ND, DP, PA, AK, KD, DP, PV, VI, IL, LW, WL, LN, NG, GG, GP, PG, GC, CS, SS, SL, LT, TG, GL as reading sequence from left to right. Protein II is converted to 29 adjacent pairs that are WF, FF, FE, ES, SR, RN, ND, DP, PA, AN, ND, DP, PI, II, IL, LW, WL, LN, NG, GG, GP, PG, GC, CS, SS, SF, FT, TG, GL as reading sequence from left to right. For example, “ND” pair has a count one in protein I and two in protein II. “DP” pair has a count two in both protein I and protein II. “SL” and “LT” pairs have a count one in protein I and zero in protein II.

Our approach is applied on three selected groups of protein sequences. The groups are beta globin, ND5, and spike protein sequences as illustrated in Tables 1, 2, and 3, respectively. The most common protein sequences are selected in each group. The selected sample is assumed to be unbiased and the population distribution of each group is normal. Therefore, the selected three samples can represent the three groups. The samples consist of seven beta globin, nine ND5, and 29 spike protein sequences.

Seven adjacency vectors for beta globin proteins, nine adjacency vectors for ND5 protein sequences, and 29 adjacency vectors for spike proteins are evaluated. For example:

 (1) Human (beta globin) protein sequence's first 20 elements of its adjacency vector (A_human  beta  globin_) are as shown in Table 4.
 (2) Gorilla (ND5) protein sequence's last 20 elements of its adjacency vector (A_gorilla  ND5_) are as shown in Table 5.

## 4. The Group Representative Vector

The adjacency vector is used to describe each protein sequence individually in its corresponding group. This article provides a descriptor to the group itself. The median vector is selected to play the role of the group representative (*GR*_*y*_);* y* refers to its group. It acts as a reference vector for each group. The median is a better measure of central tendency. It separates the higher half from the lower half of the sample's data. It is not sensitive to extreme values like average.

The suggested group representative vector (*GR*_*y*_) is a vector which is composed of also 400 elements. Each element of 400 is the median of the corresponding elements in all adjacency vectors related to its sample that represents the group. Beta globin, ND5, and spike protein sequences' representative vectors are computed. For example:

  (1) Beta globin representative vector's (GR_beta  globin_) 1^st^ 20 elements are as shown in Table 6.
  (2) ND5 representative vector's (GR_ND5_) last 20 elements are as shown in Table 7.
  (3) Spike proteins representative vector's (GR_spike  proteins_) 1^st^ 20 elements are as shown in Table 8.

## 5. Similarity/Dissimilarity Analysis

A similarity/dissimilarity vector is introduced instead of the regular similarity/dissimilarity matrix [10, 11]. The similarity/dissimilarity matrix is a square symmetric matrix with zeros in its main diagonal. In order to evaluate this matrix, it is required to measure the degree of similarity between each protein sequence and others in the same group. If the 1^st^ row represents human and the 2^nd^ row represents gorilla, the similarity of all species according to human in 1^st^ row is measured. Then the similarity is measured again of all species in 2^nd^ row according to gorilla and so on. The calculations' number of this matrix equals ∑_*k*=*n*_^1^(K − 1)/2 where n is the number of compared species.

The similarity/dissimilarity vector is suggested to save time and number of calculations. It is a vector that has a number of elements equal to the number of protein sequences in the selected sample of each group. It measures the degree of similarity between each protein sequence's adjacency vector and the group representative vector. In other words, it measures the degree of similarity between each protein's descriptor and the “group representative.” It is simpler than previous matrix. It is calculated only one time for each sequence. The calculations' number of this vector equals n where n is the number of compared species.

To measure the degree of similarity, we suggest two methods:

*(i) The 1* ^*st*^ *Method*. Evaluate the magnitude of the difference between each protein sequence' adjacency vector (*A*_*xy*_) and the group representative vector (*GR*_*y*_) of its sample as in

(1) Dxy=Axy−GRywhere:  a,b,c,d=a2+b2+c2+d2

*(ii) The 2* ^*nd*^ *Method*. Compute the angle between each sequence's adjacency vector (*A*_*xy*_) and the group representative vector (*GR*_*y*_) in radians by

(2) $$\begin{array}{c} \theta_{xy}={cos}^{-1}\left[\;\frac{\left(A_{xy}\cdot GR_{y}\right)}{\left(\left\|A_{xy}\right\|\times \left\|GR_{y}\right\|\right)}\;\right] \end{array}$$

For beta globin protein sequences, seven species are selected in our sample set: human, chimpanzee, gorilla, mouse, rat, gallus, and opossum, as illustrated in Table 1. There are seven adjacency vectors corresponding to them. The group representative GR_beta  globin_ is evaluated based on these seven adjacency vectors. Therefore, the similarity/dissimilarity vector has seven elements. The 1^st^ element corresponds to human, 2^nd^ element corresponds to chimpanzee, and so on, by the same order as in Table 1. In the similar manner, the ND5 similarity/dissimilarity vector and the 29 spike similarity/dissimilarity vector have nine elements and 29 elements as shown in Tables 2 and 3, respectively.

The similarity/dissimilarity vectors that are corresponding to beta globin, ND5, and spike protein sequences are illustrated in Tables 9, 10, and 11, respectively, based on the two methods discussed before.

**Figure 1:**
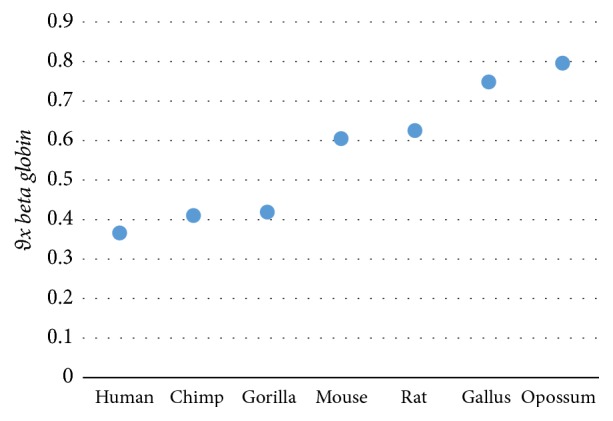
Similarity/dissimilarity analysis results of 7 beta globin protein sequences based on *θ*_*x*  *beta*  *globin*_.

The results in Table 9 show that the magnitude (*D*_*x*  *beta*  *globin*_, where* x*: species) cannot measure the similarity/dissimilarity degree well among all beta globin sequences. The human, chimpanzee, and gorilla have the same value that is equal to 0.5568, while the similarity is well measured between mouse and rat. Also, the dissimilarity between opossum and human is very clear. The angle (*θ*_*x*  *beta*  *globin*_) is successfully measured similarity/dissimilarity among all the species as shown in Figure 1. The closest values of both *D*_*x*  *beta*  *globin*_ and *θ*_*x*  *beta*  *globin*_ mean more similarity.

**Figure 2:**
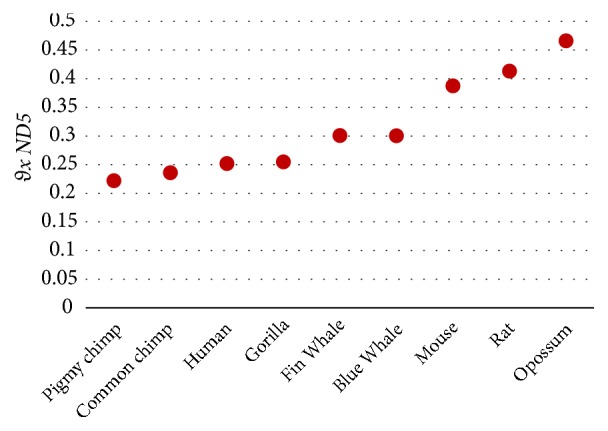
Similarity/dissimilarity analysis results of 9 ND5 protein sequences based on *θ*_*x*  *ND*5_.

The results in Table 10 show that both the magnitude (*D*_*x*  *ND*5_) and the angle (*θ*_*x*  *ND*5_) can measure similarity/dissimilarity degree well among ND5 protein sequences as shown in Figure 2. It is obvious that pigmy chimpanzee, common chimpanzee, human, and gorilla are very similar. Also it shows the similarity of the blue whale, fin whale, and the mouse and rat as pairs and the dissimilarity between human and opossum. These results are satisfied with [13, 14, 16, 18, 19, 21–25].

**Figure 3:**
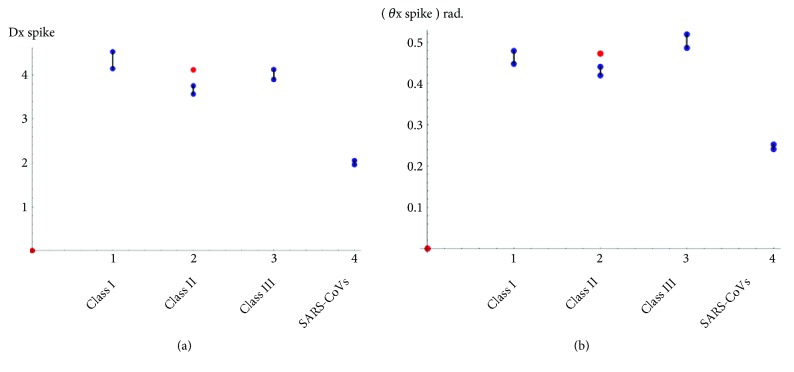
Similarity/dissimilarity analysis results of 29 spike protein sequences (a) based on *D*_*x*  *spike*_ (b) based on *θ*_*x*  *spike*_.

The results in Table 11 show that both *D*_*x*  *spike*_ and *θ*_*x*  *spike*_ classified the 3 classes of viruses and SARs_Covs well each as a single coherent class except only the “MHVJHM” virus. This virus belongs to class II but our approach cannot classify it well. The classification of 29 spike proteins into classes by our approach is illustrated in Figure 3. The MHVJHM virus is the only wrong classified sequence. It is colored red. Despite the wrong classification of MHVJHM virus, our approach corrects the broken classification of Class I in [26].

According to the results in Tables 9, 10, and 11, the angle *θ*_*xy*_ is preferred to be used as shown in Figures 1, 2, and 3.

## 6. Cross-Group Comparison

**Figure 4:**
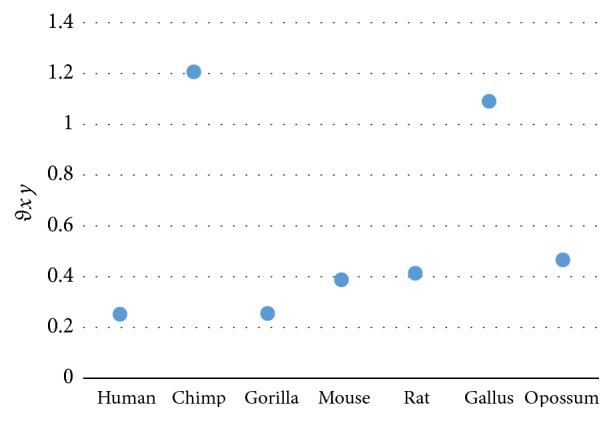
Similarity/dissimilarity analysis results of 7 beta globin protein sequences based on (GR_ND5_) (*θ*_*xy*_).

**Figure 5:**
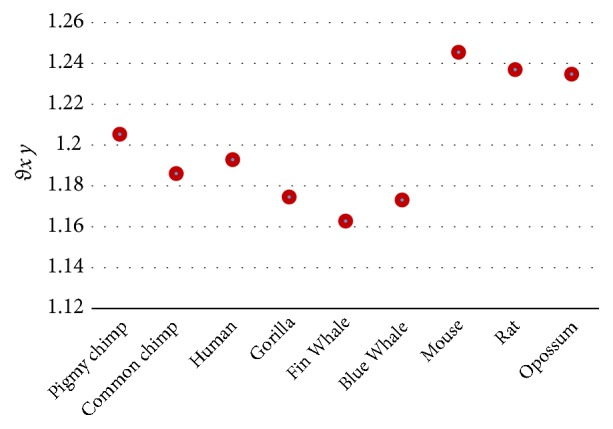
Similarity/dissimilarity analysis results of 9 ND5 protein sequences based on (GR_beta  globin_) (*θ*_*xy*_).

**Figure 6:**
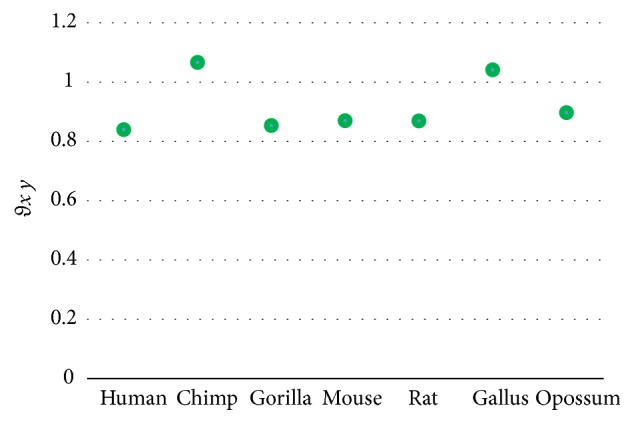
Similarity/dissimilarity analysis results of 7 beta globin protein sequences based on (GR_spike_) (*θ*_*xy*_).

**Figure 7:**
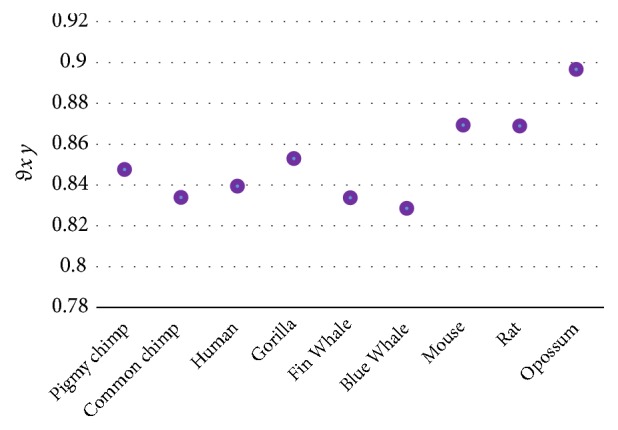
Similarity/dissimilarity analysis results of 9 ND5 protein sequences based on (GR_spike_) (*θ*_*xy*_).

The group representative vector (*GR*_*y*_) carries the information of its group. A cross-group comparison is done to prove the singularity of each group. Tables 9, 10, and 11 are evaluated based on the group's sample set of protein sequences related to their corresponding group representative vector. Tables 12, 13, 14, and 15 are evaluated based on each group sample set of protein sequences with another group representative vector. The similarity/dissimilarity analysis among the seven beta globin sequences measured according to (*GR*_*ND*5_) is illustrated in Table 12 and shown in Figure 4. The similarity/dissimilarity analysis among the ND5 sequences measured according to (*GR*_*beta*  *globin*_) is illustrated in Table 13 and shown in Figure 5. The similarity/dissimilarity analysis among the beta globin sequences measured according to (*GR*_*spike*_) is illustrated in Table 14 and shown in Figure 6. The similarity/dissimilarity analysis among the ND5 sequences measured according to (*GR*_*spike*_) is illustrated in Table 15 and shown in Figure 7. The results show a big distortion that ensures the individuality of each group.

## 7. A Qualitative Comparison between Our Results and the Phylogenetic Tree of Protein Sequences

The phylogenetic tree is a branching diagram showing the evolutionary relationships among various biological species based upon similarities and differences in their sequences. A qualitative comparison between our results and the phylogenetic tree of protein sequences is used to prove the utility of our approach. The matching between the results and phylogenetic trees means matching with the naïve measure of sequence similarity (sequence homology).

**Figure 8:**
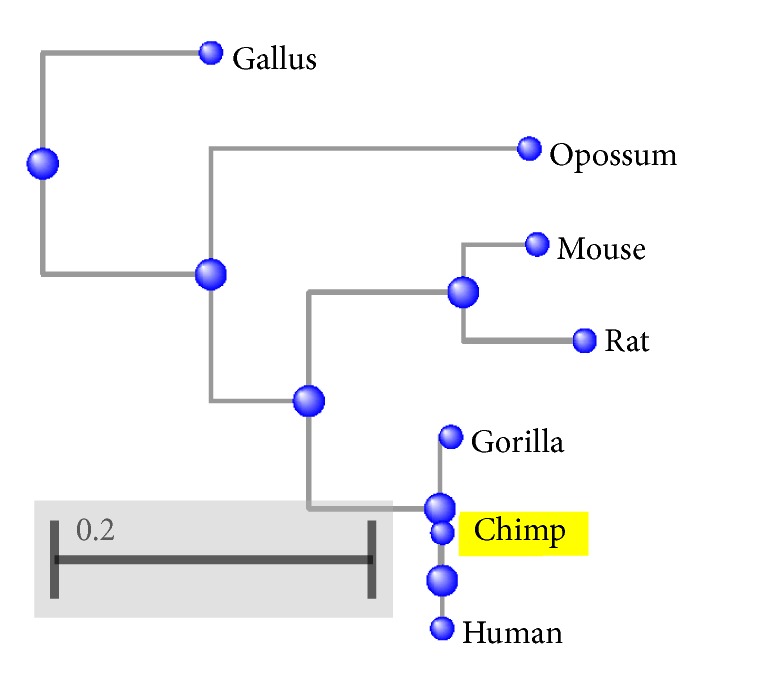
The phylogenetic tree of beta globin selected protein sequences by BLAST program.

**Figure 9:**
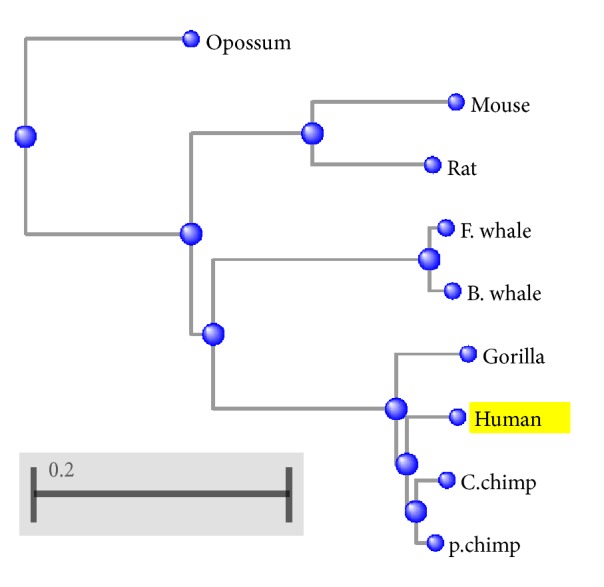
The phylogenetic tree of ND5 selected protein sequences by BLAST program.

**Figure 10:**
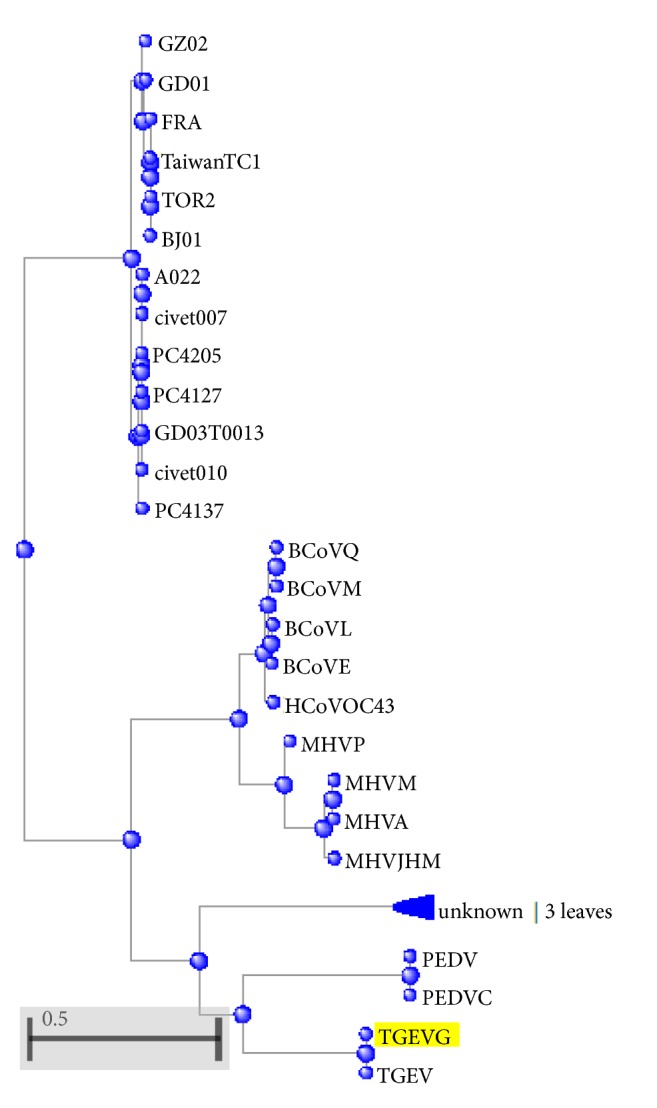
The phylogenetic tree of 29 spike protein sequences by BLAST program, 3 unknown leaves are for class III (IBVBJ, IBVC, and IBV: the tool cannot detect their names).

The basic local alignment tool (BLAST) is used to draw the phylogenetic trees. The phylogenetic trees of beta globin's seven species, ND5 nine species, and 29 spike protein sequences are illustrated in Figures 8, 9, and 10, respectively.

The qualitative comparison of the results of Tables 9, 10, and 11 and Figures 8, 9, and 10 shows the utility of our work especially the angle *θ*_*x*_ results.

## 8. Conclusion

The proposed method is an alignment-independent method. An adjacency vector is suggested as a descriptor of any protein sequence. It does not require any graphical representation. A group representative vector is introduced to represent each group of protein sequences. A similarity/dissimilarity vector is produced instead of the regular similarity/dissimilarity matrix. The similarity/dissimilarity analysis is done by two methods. Our approach is applied on three sample sets of three groups of protein sequences. Each sample has a different range of lengths than the others. Our approach does not depend on protein sequence length. It successfully measured similarity/dissimilarity among different lengths. It is very mathematically simple. A cross-grouping comparison is introduced to prove the singularity of each group. The results approved the utility of our approach compared with previous articles and phylogenetic tree obtained by BLAST program.

## 9. Future Work

We hope to make the method available to include ambiguous amino acid residues and nonstandard amino acids. We hope also to include the analyses of partial or gapped sequences.

## Figures and Tables

**Table 1:** The basic information of seven beta globin protein sequences.

No.	Species	Access No.	Length
1	Human	AAA16334	147
2	Chimpanzee	CAA26204	125
3	Gorilla	CAA43421	121
4	Mouse	CAA24101	147
5	Rat	CAA29887	147
6	Gallus	CAA23700	147
7	Opossum	AAA30976	147

**Table 2:** The basic information of nine ND5 protein sequences.

No.	Species	Access No.	Length
1	Human	AP_000649	603
2	Gorilla	NP_008222	603
3	Pigmy Chimpanzee	NP_008209	603
4	Common Chimpanzee	NP_008196	603
5	Fin Whale	NP_006899	606
6	Blue Whale	NP_007066	606
7	Rat	AP_004902	610
8	Mouse	NP_904338	607
9	Opossum	NP_007105	602

**Table 3:** The basic information of 29 spike protein sequences.

No.	Access No.	Class No.	Abbreviation	Length
1	CAB91145	I	TGEVG	1447
2	NP058424	I	TGEV	1447
3	AAK38656	I	PEDVC	1383
4	NP598310	I	PEDV	1383
5	NP937950	II	HCoVOC43	1361
6	AAK83356	II	BCoVE	1363
7	AAL57308	II	BCoVL	1363
8	AAA66399	II	BCoVM	1363
9	AAL40400	II	BCoVQ	1363
10	AAB86819	II	MHVA	1324
11	YP 209233	II	MHVJHM	1376
12	AAF69334	II	MHVP	1321
13	AAF69344	II	MHVM	1324
14	AAP92675	III	IBVBJ	1169
15	AAS00080	III	IBVC	1169
16	NP 040831	III	IBV	1162
17	AAS10463	SARS_CoVs	GD03T0013	1255
18	AAU93318	SARS_CoVs	PC4127	1255
19	AAV49720	SARS_CoVs	PC4137	1255
20	AAU93319	SARS_CoVs	PC4205	1255
21	AAU04646	SARS_CoVs	civet007	1255
22	AAU04649	SARS_CoVs	civet010	1255
23	AAV91631	SARS_CoVs	A022	1255
24	AAP51227	SARS_CoVs	GD01	1255
25	AAS00003	SARS_CoVs	GZ02	1255
26	AAP30030	SARS_CoVs	BJ01	1255
27	AAP50485	SARS_CoVs	FRA	1255
28	AAP41037	SARS_CoVs	TOR2	1255
29	AAQ01597	SARS_CoVs	TaiwanTC1	1255

**Table 4:** 

AA	AR	AN	AD	AC	AQ	AE	AG	AH	AI	AL	AK	AM	AF	AP	AS	AT	AW	AY	AV
1	0	1	0	0	0	0	1	4	0	3	0	0	1	0	0	1	0	1	2

**Table 5:** 

VA	VR	VN	VD	VC	VQ	VE	VG	VH	VI	VL	VK	VM	VF	VP	VS	VT	VW	VY	VV
5	0	0	0	1	1	0	1	0	0	0	1	0	3	1	1	2	0	0	1

**Table 6:** 

AA	AR	AN	AD	AC	AQ	AE	AG	AH	AI	AL	AK	AM	AF	AP	AS	AT	AW	AY	AV
1	0	0	0	0	0	1	1	3	0	2	0	0	1	0	0	1	0	0	2

**Table 7:** 

VA	VR	VN	VD	VC	VQ	VE	VG	VH	VI	VL	VK	VM	VF	VP	VS	VT	VW	VY	VV
2.5	0	0	0	0	0	0	1	0	0	0.5	1	0	3.5	0	1	3.5	0	0	1

**Table 8:** 

AA	AR	AN	AD	AC	AQ	AE	AG	AH	AI	AL	AK	AM	AF	AP	AS	AT	AW	AY	AV
9	1	3	5	2	4	3	6	0	7	6	2	1	3	6	4	7	1	5	3

**Table 9:** Similarity/dissimilarity vector among 7 different species of beta globin protein sequences.

No.	Species	*D*_*x* *beta* *globin*_	(Θ_*x* *beta* *globin*_) rad.
1	Human	0.5568	0.3657
2	Chimpanzee	0.5568	0.4098
3	Gorilla	0.5568	0.4185
4	Mouse	0.8602	0.6047
5	Rat	0.9165	0.6251
6	Gallus	1.0536	0.7480
7	Opossum	1.1136	0.7955

**Table 10:** Similarity/dissimilarity vector among 9 different species of ND5 protein sequences.

No.	Species	*D*_*x* *ND*5_	(Θ_*x* *ND*5_) rad.
1	Pigmy chimpanzee	1.2530	0.2218
2	Common chimpanzee	1.3191	0.2357
3	Human	1.3856	0.2517
4	Gorilla	1.3892	0.2547
5	Fin Whale	1.5395	0.3006
6	Blue Whale	1.5459	0.3003
7	Mouse	2.0372	0.3873
8	Rat	2.1517	0.4130
9	Opossum	2.3367	0.4659

**Table 11:** Similarity/dissimilarity vector among 29 different species of spike protein sequences.

	Abbreviation	Class no.	*D*_*x* *spike*_	(*θ*_*x* *spike*_*) rad.*
1	TGEVG	I	4.5266	0.4793
2	TGEV	I	4.5266	0.4793
3	PEDVC	I	4.1413	0.4473
4	PEDV	I	4.1413	0.4473
5	HCoVOC43	II	3.7537	0.4299
6	BCoVE	II	3.7377	0.4203
7	BCoVL	II	3.7550	0.4233
8	BCoVM	II	3.7216	0.4198
9	BCoVQ	II	3.7216	0.4203
10	MHVA	II	3.7095	0.4395
*11*	*MHVJHM*	*II*	*4.1183*	*0.4728*
12	MHVP	II	3.5651	0.4240
13	MHVM	II	3.7014	0.4406
14	BVBJ	III	3.9699	0.5002
15	IBVC	III	3.8936	0.4863
16	IBV	III	4.1243	0.5188
17	GD03T0013	SARS-CoVs	1.9824	0.2439
18	PC4127	SARS-CoVs	2.0075	0.2473
19	PC4137	SARS-CoVs	2.0224	0.2491
20	PC4205	SARS-CoVs	2.0099	0.2476
21	civet007	SARS-CoVs	2.0469	0.2519
22	civet010	SARS-CoVs	2.0125	0.2478
23	A022	SARS-CoVs	2.0518	0.2526
24	GD01	SARS-CoVs	1.9824	0.2445
25	GZ02	SARS-CoVs	1.9723	0.2433
26	BJ01	SARS-CoVs	1.9570	0.2413
27	FRA	SARS-CoVs	2.0125	0.2481
28	TOR2	SARS-CoVs	1.9949	0.2458
29	TaiwanTC1	SARS-CoVs	1.9875	0.2449

**Table 12:** Similarity/dissimilarity vector among 7 different species of beta globin protein sequences according to (*GR*_*ND*5_).

No.	Species	Dxy	*θ*xy
1	Human	1.38564	0.251674
2	Chimp	4.71593	1.20638
3	Gorilla	1.38924	0.254656
4	Mouse	2.03715	0.387323
5	Rat	2.15174	0.41301
6	Gallus	4.53211	1.08994
7	Opossum	2.33666	0.465884

**Table 13:** Similarity/dissimilarity vector among 9 different species of ND5 protein sequences according to (*GR*_*beta*  *globin*_).

No.	Species	Dxy	*θ*xy
1	Pigmy chimp	5.16914	1.20525
2	Common chimp	5.14101	1.18598
3	Human	5.12348	1.19282
4	Gorilla	5.07346	1.1745
5	Fin whale	4.82286	1.16274
6	Blue whale	4.86621	1.17307
7	Mouse	5.12445	1.2454
8	Rat	5.07346	1.23689
9	Opossum	4.81768	1.23466

**Table 14:** Similarity/dissimilarity vector among 7 different species of beta globin protein sequences according to (*GR*_*spike*_).

No.	Species	Dxy	*θ*xy
1	Human	6.02661	0.839369
2	Chimp	7.52463	1.06606
3	Gorilla	6.1	0.852902
4	Mouse	6.18789	0.869323
5	Rat	6.18466	0.8689
6	Gallus	7.44849	1.04124
7	Opossum	6.32614	0.896635

**Table 15:** Similarity/dissimilarity vector among 9 different species of ND5 protein sequences according to (*GR*_*spike*_).

No.	Species	Dxy	*θ*xy
1	Pigmy chimp	6.07207	0.847581
2	Common chimp	5.99667	0.833859
3	Human	6.02661	0.839369
4	Gorilla	6.1	0.852902
5	Fin whale	6.00083	0.833717
6	Blue whale	5.97244	0.828506
7	Mouse	6.18789	0.869323
8	Rat	6.18466	0.8689
9	Opossum	6.32614	0.896635

## Data Availability

All data is mentioned clearly in the manuscript in Section 2 under the title “Dataset.” In this section, we illustrate the data in three tables: Tables 1, 2, and 3. We also mention in the 1st paragraph of dataset that data are downloaded from “Gene Bank.” All data files are with extension “. fasta”.

## Supplementary Materials

Supplementary Materials It is a figure which summarizes our approach. It is submitted under the name of Graphical Abstract.
