# Supplementary figures and images for: A Statistical Similarity/Dissimilarity Analysis of Protein Sequences Based on a Novel Group Representative Vector
Source: Biomed Res Int. 2019 May 8;2019:8702968. doi: 10.1155/2019/8702968 (PMC6530227; doi:10.1155/2019/8702968)

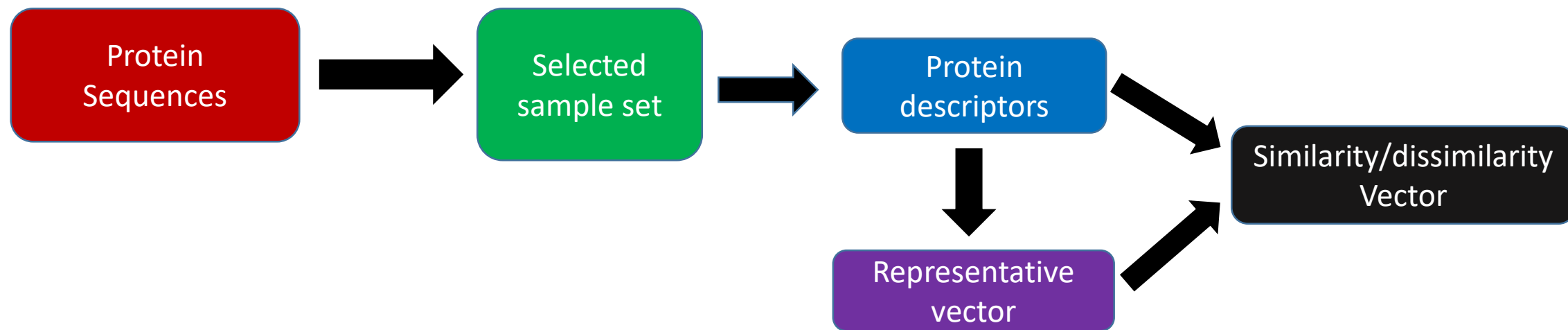

Supplement: Supplementary Materials — It is a figure which summarizes our approach. It is submitted under the name of Graphical Abstract. [file 8702968.f1.pdf]
